# Scaling up natural experimental studies: harnessing emerging technologies to transform physical activity and built environment research

**DOI:** 10.1186/s12966-025-01742-7

**Published:** 2025-04-11

**Authors:** Jack S. Benton, Marilyn E. Wende, Declan J. Ryan, Rachel L. Thompson, J. Aaron Hipp

**Affiliations:** 1 Department of Geography, School of Environment, Education and Development, The University of Manchester, Manchester, UK; 2 Department of Health Education and Behavior, College of Health and Human Performance, University of Florida, Gainesville, FL USA; 3 Centre for Physical Activity and Life Sciences, University of Northampton, Northampton, UK; 4 Department of Environmental, Occupational, and Geospatial Health Sciences, Graduate School of Public Health and Health Policy, City University of New York, New York, NY USA; 5 Department of Parks, Recreation, and Tourism Management, College of Natural Resources, NC State University, Raleigh, NC USA

**Keywords:** Built environment, Natural experiments, Physical activity, Measurement, Emerging technology

## Abstract

Evaluating how built environment interventions influence physical activity is crucial for informing effective policies. Natural experiments provide opportunities to assess real-world interventions, yet traditional data collection methods - such as surveys, accelerometers, manual observation and environmental audit tools - limit scalability due to inefficiencies, low participant engagement, and biases. As a result, there is a lack of robust, generalisable evidence to inform policymakers on the most effective built environment interventions for promoting physical activity across diverse communities. In this commentary, we outline four emerging technologies that could address these challenges: (1) smartphone applications and wearable technology for physical activity measurement, (2) geolocation data for assessing mobility patterns, (3) automated systematic observation of physical activity behaviours, and (4) automated environmental audits. We discuss how these approaches can enhance the scalability of natural experimental studies while also considering important ethical implications, including privacy, inclusivity, and community engagement. Advancing and integrating these technologies is critical for generating robust evidence to design built environments that equitably support physical activity.

Improving the built environment is now well-recognised as a key population-level intervention for promoting physical activity (PA) [1]. Promising interventions include enhancing green spaces, improving walkability, and creating low-traffic neighbourhoods. Since researchers do not control these changes, natural experiments (i.e., real world interventions outside researchers’ control) are ideal for evaluating the causal effects of built environment interventions on PA [2]. Although definitions vary, the United Kingdom (UK) Medical Research Council (MRC) guidance defines natural experiments as “events, interventions or policies which are not under the control of researchers, but which are amenable to research which uses the variation in exposure that they generate to analyse their impact” [ [3]: p.4]. Researchers can therefore design studies around a natural experiment to evaluate the impact of such interventions on PA, hereafter referred to as natural experimental studies.

Despite the growing use of natural experiments, researchers often still rely on traditional data collection methods such as surveys, accelerometer devices, manual observations, or manual audits of the built environment. These methods limit our ability to conduct large-scale natural experimental studies due to inefficiencies (e.g., resource-intensive data collection, time-consuming manual coding, difficulty in capturing long-term trends), biases (e.g., recall, reactivity, sampling), and low participant engagement. Although routinely collected surveillance data can be more scalable, there are challenges related to inconsistent data quality and limited assessment of environmental exposure. As a result, there remains a shortage of robust, generalisable evidence to inform policymakers on which built environment interventions are most effective for promoting PA across diverse communities.

To address this, there is an urgent need for more scalable methods for measuring PA and the built environment. This commentary outlines four promising avenues where emerging technologies can be leveraged to enhance both the scalability and rigour of natural experimental research in the PA field.

## Smartphone applications and wearable technology for measuring physical activity

Smartphones provide a scalable platform for studying large-scale population health outcomes due to their widespread use in developed countries [4]. These devices can continuously track multiple PA metrics, including step counts, distance travelled, and active minutes. For example, a recent study tracking the step counts of 455,404 users of a free smartphone app during the COVID-19 pandemic demonstrated its potential for large-scale PA monitoring [5]. Moreover, the expanding market for smartphone-pairable fitness trackers and smartwatches with advanced sensors (e.g., gyroscopes, accelerometers) enables more detailed PA analyses and data harmonisation. These PA data, when combined with GPS information, can provide place-based insights into PA patterns.

However, smartphones and wearables vary in their ability to accurately characterise PA. A review of 25 studies found mixed evidence on the accuracy of various smartphone apps for tracking steps, distance, and energy expenditure [6]. Some wrist-wearable activity trackers have demonstrated better accuracy [7]. Future research should therefore focus on large-scale validation studies of smartphone apps and wearables across diverse populations, addressing issues such as device placement, environmental conditions, and user adherence.

## Geolocation data for assessing population mobility patterns

Geolocation data from GPS-enabled smartphones offers a promising solution for monitoring real-time population mobility patterns at high spatial and temporal resolution. This method is particularly useful for distinguishing true behavioural changes from displacement effects - a key challenge in evaluating interventions like active travel schemes, where increases in pedestrians or cyclists may reflect shifts in location rather than genuine changes in travel mode [8].

Companies such as ActiveXchange [9] and Place Informatics [10] are enhancing this method by sourcing anonymised GPS data from tens of thousands of apps and hundreds of millions of devices. Since users typically provide consent once during app installation– which often includes permission for data sharing and third-party use– this method minimises participant burden while yielding granular insights into site usage and movement patterns. Importantly, access to historical data helps address the challenge of establishing baseline measures in natural experimental studies.

Although geolocation data do not support individual-level comparisons, they can estimate the geographic areas (e.g., postcodes or lower-layer super output areas) where smartphone users reside, which enables researchers to track which communities access specific locations over time. However, commercial decisions can affect data quality and availability. For example, Placer.ai [11] recently removed playgrounds and schools from its dataset for privacy reasons, highlighting the evolving nature of commercial geolocation data. More research is needed to assess the validity of geolocation data (e.g., location accuracy), particularly for underrepresented groups (e.g., children) and areas with low smartphone use (e.g., isolated rural locations).

## AI for automating systematic observation of physical activity behaviour

Advancements in video surveillance and artificial intelligence (AI) offer new opportunities to scale up traditional, labour-intensive observation tools such as the System for Observing Play and Recreation in Communities (SOPARC) [12]. High-resolution video cameras can capture recordings in public spaces, which can then be analysed using deep learning models (a subset of machine learning) to detect, track, and quantify PA. Once trained, AI-based observational methods could substantially reduce the labour, time, safety risks, and costs associated with data collection, enabling large-scale data acquisition over extended periods and across diverse settings. AI’s capability for real-time analysis may allow for immediate feedback on PA outcomes linked to environmental interventions, thereby supporting timely, data-driven decision-making by urban planners and public health officials.

Several companies (e.g., Miovision [13]) already offer video-based pedestrian tracking solutions, and emerging research is beginning to establish the accuracy of these AI models [14]. However, further research is needed to assess performance under varying conditions (e.g., in densely built urban environments) and to explore whether AI can reliably distinguish between PA intensities (e.g., moderate vs. vigorous activity).

## AI for automating built environment audits

AI can also transform traditional environmental audits, which are crucial for understanding how built environments influence PA [15] but are often expensive, time-consuming, and pose safety risks for auditors. By applying computer vision techniques to ground-level imagery (e.g., Google Street View [16]) and aerial drone images [17], researchers can automatically detect, quantify and monitor environmental features over large geographical areas. This could enable large-scale, longitudinal epidemiological assessments of how changes in both macro- and microscale features of the built environment impact PA and health outcomes. Automated environmental audits can also help identify appropriately matched control sites in natural experimental studies by comparing specific environmental features between intervention and non-intervention areas.

Automated environmental audits can also enhance the generalisability of natural experimental studies by allowing researchers to assess changes in pedestrian environments across diverse geographic and socio-demographic contexts, including vast rural areas that are difficult to audit manually. However, while automated environmental audits enable the analysis of thousands of locations simultaneously, most existing AI-based tools have been developed and validated primarily in urban settings. To maximise their potential, these computer vision models must be refined to ensure robust performance across a wider range of environments, including both urban and rural areas worldwide.

## Ethical considerations

If we are to harness these emerging technologies to improve the scalability of natural experimental studies, it is essential to consider the ethical implications surrounding their use, particularly regarding privacy, consent, and data security. Geolocation data, for example, can reveal detailed movement patterns, which may raise concerns about potential misuse, unauthorised access, and privacy violations. It is therefore essential that data management practices adhere to rigorous ethical and legislative standards, which can vary substantially by country and institution.

Access to valuable datasets - including footfall, mobility, and AI-detected environmental data– is often controlled by private companies, which may impose significant costs for access. This raises ethical concerns about the commercialisation of data that could otherwise be used to improve public health. To ensure these benefits are widely accessible, it is crucial to establish fair and equitable data sharing practices.

Biases in AI systems - whether racial, gender, or related to other factors - can perpetuate inequalities if not actively addressed. Additionally, disparities in smartphone access and network coverage can limit the inclusivity of smartphone-based research, particularly in rural or low-income areas. We must continuously evaluate and rectify these biases to minimise risks of biased decision-making that disproportionately benefit populations with better access to such technology.

Finally, community engagement is fundamental, particularly when working with vulnerable or under-resourced groups. Communities with historical experiences of structural racism or institutional mistrust may perceive technologies, such as video surveillance, as an invasion of privacy or a form of colonial science. Meaningfully engaging communities from the start of research projects can foster trust and ensures that their concerns are being heard and addressed.

## Conclusions

A major shift in research practices is needed to scale up natural experimental studies on how changes to the built environment influence PA. Embracing emerging technologies that are more efficient and scalable than traditional methods is essential for generating the robust evidence needed to drive meaningful change. We outline four promising avenues that could rapidly advance our understanding of how to modify built environments to promote PA, along with important ethical considerations for their use. Interdisciplinary collaboration will be key to harnessing these technologies, and the potential for international partnerships through scalable technologies presents exciting opportunities for global progress.

## Data Availability

No datasets were generated or analysed during the current study.

## Abbreviations

AI: Artificial Intelligence
COVID-19: Coronavirus Disease 2019
GPS: Global Positioning System
PA: Physical Activity
SOPARC: System for Observing Play and Recreation in Communities
